# Impaired antioxidant defense associated with flavonoid biosynthesis reprogramming in the mangrove *Acanthus ilicifolius* under high salinity

**DOI:** 10.3389/fpls.2026.1853520

**Published:** 2026-08-05

**Authors:** Qingfan Xiong, Zhengzheng Sun, Lin Su, Yilai You, Chao Chen

**Affiliations:** 1 Zhuhai Qi’ao-Dangan Island Provincial Nature Reserve Management Office, Zhuhai, China; 2 Instrumentation and Service Center for Science and Technology, Beijing Normal University at Zhuhai, Zhuhai, China

**Keywords:** *Acanthus ilicifolius*, salinity stress, mangrove plant, oxidative stress, metabolomics, transcriptomics, flavonoid biosynthesis, salt tolerance

## Abstract

**Introduction:**

This study investigates how varying salinity levels influence the physiological and metabolic responses of the mangrove plant *Acanthus ilicifolius*, aiming to clarify its salt adaptation mechanisms.

**Methods:**

Field experiments were conducted at three sites with stable salinity conditions (approximately 0.73, 2.26, and 3.09 g/L), combined with physiological assays, metabolomics, and transcriptomics.

**Results:**

Increasing salinity elevated intracellular reactive oxygen species, reduced antioxidant enzyme activity, and aggravated membrane lipid peroxidation. Metabolomics revealed that medium salinity primarily affected terpenoid (18.7%) and lipid (14.5%) metabolism, while high salinity regulated flavonoid (11%) and terpenoid (16.4%) biosynthesis. Transcriptomic data indicated that the antioxidant defense system plays a central role in mitigating oxidative stress, with WRKY and AP2/ERF transcription factors significantly upregulated under high salinity, enhancing kaempferol-flavanone isomerase activity and flavonoid accumulation (e.g., hesperidin).

**Discussion:**

These findings show that *A. ilicifolius* compensates for impaired antioxidant defenses by redirecting metabolism toward flavonoid synthesis, providing novel insights into the molecular basis of mangrove salt tolerance and its potential medicinal applications.

## 1 Introduction

*A. ilicifolius* is a typical mangrove-associated species that inhabits intertidal ecosystems, where multiple abiotic stressors, including high salinity, periodic flooding, elevated temperature, and intense ultraviolet radiation, act simultaneously (Feng et al., 2024). These harsh environmental conditions impose severe constraints on plant survival, primarily through salt-induced ionic imbalance and osmotic stress, which subsequently trigger excessive accumulation of reactive oxygen species (ROS) and cellular damage. Compared with terrestrial plants, mangrove and mangrove-associated species such as *A. ilicifolius* have evolved efficient antioxidant defense systems to mitigate oxidative stress under saline environments. In general, plants rely on a coordinated antioxidant network to cope with salt-induced oxidative injury (Maryum et al., 2022; Zhou et al., 2024). This network mainly includes two categories of components: (1) antioxidant enzymes, such as superoxide dismutase (SOD), catalase (CAT), ascorbate peroxidase (APX), and polyphenol oxidase (POX); and (2) non-enzymatic antioxidants, including glutathione, ascorbic acid, polyphenolic compounds, flavonoids, terpenoids, and coumarins. Together, these components scavenge ROS and contribute substantially to the overall antioxidant capacity of plants (Azeem et al., 2023; Du et al., 2024; Wang et al., 2024; Zhu et al., 2025). Among them, polyphenols and flavonoids are particularly important because they act as effective electron donors, interrupt oxidative chain reactions, and help preserve the integrity of macromolecules and cellular membranes under stress conditions (Horemans et al., 2000; Zahra et al., 2024). These observations suggest that the secondary metabolites produced by plants in extreme habitats may have important biological activities.Despite its ecological importance, the physiological and molecular mechanisms underlying salt tolerance in *A. ilicifolius* remain insufficiently understood. Yang et al. reported that *A. ilicifolius* possesses at least 99 positively selected genes, 23 of which are associated with tolerance to salinity, heat, and ultraviolet stress, as well as seed germination and embryonic development under periodic flooding conditions (Yang et al., 2015). In addition, Liu and Zheng found that flooding stress maintained morphological integrity in *A. ilicifolius*, increased cellular energy charge and the soluble sugar-to-starch ratio, and enhanced the activities of several antioxidant-related proteins, including L-ascorbate peroxidase, thioredoxin, monodehydroascorbate reductase, and CAT (Liu and Zheng, 2021). However, a systematic understanding of how *A. ilicifolius* redox homeostasis and antioxidant defense at multiple molecular levels under salt stress is still lacking.

To address this gap, the present study investigated the molecular basis of salt tolerance in *A. ilicifolius* by integrating transcriptomic and metabolomic analyses under different salinity conditions. Through this multi-omics strategy, we systematically characterized the redox regulatory network and antioxidant defense mechanisms involved in the salt-stress response of *A. ilicifolius*. To the best of our knowledge, this is the first comprehensive multi-omics analysis of *A. ilicifolius* under varying salinity treatments. Our findings provide new insight into the adaptive mechanisms of this species in saline environments.

## 2 Materials and methods

### 2.1 Sampling sites and plant material

Three distinct sites with anticipated various salinity gradients were selected within the mangrove forest ecosystem of Qiao Island (22.427430° N, 113.629040° E), China, for sampling purposes. To assess salinity stability at these locations, systematic measurements were conducted across all three sites over a five-day period using Time Domain Reflectometry (TDR) sensors (FP/mts equipped with FOM/mts, E-Test Sp. z o.o., Poland). However, this short-term monitoring may not fully represent seasonal or tidal variability. For the sampling procedure, five consistent sampling points were established at a distance of 5 cm from the rhizosphere of *A. ilicifolius* specimens. The TDR sensor was inserted to a depth of approximately 10 cm, where bulk electrical conductivity (BEC) measurements of the soil matrix were recorded as an indicator for salinity levels. Soil water-soluble total salt content was determined using the gravimetric method. Briefly, soil samples were extracted with water, and the extract was collected after centrifugation or settling. The supernatant was transferred to a pre-weighed container and evaporated to dryness. The residue was then weighed, and the total salt content was calculated from the mass of the dried residue. BEC, salinity, and moisture content data are summarized in **Supplementary Table 1**. Linear regression was performed to evaluate the relationship between BEC and salinity, and the fitted model was used to estimate salinity values from measured BEC levels.

During field sampling, individual *A. ilicifolius* plants were selected as biological replicates only when they were spatially separated by approximately 5 m from the nearest sampled individual. Root tissues of *A. ilicifolius* were obtained 5 cm from the lignified significant root structures, with segments measuring 10 cm in length being excised. The specimens were subsequently flash-frozen with dry ice before transport to the laboratory facility, where they were maintained at -80 °C to ensure preservation for forthcoming analytical procedures.

### 2.2 Quantification of reactive oxygen species, saponin content, and antioxidant enzyme activity

The assessment of $O_{2}^{\cdot -}$ concentration, SOD, malondialdehyde (MDA), CAT, and glutathione reductase (GR) was conducted according to the manufacturer’s protocol (Suzhou Keming Biotechnology Co., Ltd.). For experimental analysis, three biological replicates were systematically collected from each sampling point, with each replicate comprising three distinct plant individuals. It should be noted that the root systems of *A. ilicifolius* potentially exhibit underground interconnections. All subsequent experimental procedures utilized plant samples consisting of three independent root systems.

### 2.3 Transcriptomic analysis and quantitative real-time polymerase chain reaction validation

RNA extraction was performed on soft root samples utilizing the RNAprep Pure Plant Kit (DP441, Tiangen, China). Subsequent RNA sequencing was conducted by Metware Biotechnology Co., Ltd. (Wuhan, China). The experimental methodology followed a systematic approach: initially, mRNA quantification and quality assessment were executed via a NanoPhotometer spectrophotometer (IMPLEN, California, USA). RNA integrity validation was accomplished through electrophoresis on a 1% agarose gel. Following these preliminary steps, cDNA libraries were established, and high-throughput sequencing was then implemented on the Illumina Novaseq 6000 platform. Quality control measures included the application of Fastp software (v0.24.1) to eliminate adapter sequences and substandard sequences containing ≥5 uncertain bases or exhibiting more than 50% of Qphred values ≤20 bases. Subsequently, Trinity software (v2.15.2) was employed for high-quality read assembly. Assembly completeness verification was performed using BUSCO software (https://busco.ezlab.org). Unigene sequence annotation involved alignment with multiple databases (KEGG, NR, Swiss-Prot, GO, COG/KOG, and Trembl) via DIAMOND (v2.1.8) and BLASTX software (NCBI BLAST 2.12.0). Following Unigene amino acid sequence prediction, further annotation information was acquired through alignment with the Pfam database using HMMER software (v3.4). TransDecoder (https://github.com/TransDecoder/) facilitated the prediction of coding sequences (CDSs) for the assembled transcripts. Finally, the Trinity-assembled, deduplicated transcripts were designated as reference sequences, to which clean reads from individual samples were aligned.

RNA-seq raw counts were analyzed using DESeq2 software (v1.48.1) for differential expression analysis, followed by functional enrichment analysis via the clusterProfiler package (v4.16.0). For Gene Ontology (GO) analysis, we implemented a hierarchical clustering approach utilizing 6–7 levels to analyze the relationships between RNA samples. The top 20 functional categories, ranked according to log *p*-adjusted values (using the Benjamini-Hochberg correction method), were selected for visualization of the GO enrichment results. For Kyoto Encyclopedia of Genes and Genomes (KEGG) pathway analysis, we employed the *Arabidopsis thaliana* reference database to comprehensively evaluate metabolic pathway alterations in *A. ilicifolius* under varying salinity gradient conditions.

Gene expression counts were aggregated within experimental groups before analysis. Differential gene expression was determined using DESeq2 with stringent filtering parameters: adjusted *p*-value< 0.05, false discovery rate (FDR) ≤ 0.01, and absolute log2 fold change (|log2FC|) ≥ 1.

Sequencing accuracy was validated through quantitative PCR (qPCR) of three randomly selected genes. Gene-specific primers were designed using Primer Premier 5 software to target the sequences of interest. Complete primer sequences are documented in **Supplementary Table 2**. The original gene sequence used for qPCR is provided in FASTA format (PCR_Unigenes.fa file). Relative quantification of gene expression was calculated using the 2^(^-ΔΔCt^) method. All experimental procedures were conducted in triplicate to ensure data reproducibility and statistical reliability.

### 2.4 Metabolite extraction, identification, and quantitative analysis

Metabolite identification and analysis were conducted by Wuhan Meite Biotechnology Co., Ltd. (Wuhan, China). Biological samples underwent vacuum freeze-drying (Scientz-100F freeze dryer) for 63 hours, followed by pulverization using a Retsch MM 400 mill (30 Hz, 1.5 minutes). Subsequently, approximately 30 mg of the resultant powder was extracted with 1,500 μL of pre-chilled 70% methanol containing internal standard (proportionally adjusted). The internal standard solution was prepared at a concentration of 250 μg/mL by dissolving the standard material in 70% methanol. Samples were subjected to intermittent vortexing (30 seconds every 30 minutes, repeated 6 times), centrifugation (12,000 rpm, 3 minutes), and filtration through a 0.22 μm membrane. The filtrate was then analyzed via UPLC-MS/MS. Chromatographic separation was achieved using an Agilent SB-C18 column (1.8 µm, 2.1 mm × 100 mm) with mobile phase A (0.1% formic acid in ultra-pure water) and mobile phase B (0.1% formic acid in acetonitrile). The gradient elution program was established as follows: 5-95% B (9 minutes), maintained at 95% B (1 minute), then returned to initial conditions. The flow rate, column temperature, and injection volume were set at 0.35 mL/min, 40 °C, and 2 μL, respectively. Mass spectrometric detection employed ESI with the following parameters: temperature (500 °C), ion spray voltage (5500 V for positive ions; -4500 V for negative ions), gas pressures (I: 50 psi, II: 60 psi, curtain: 25 psi), nitrogen as collision gas, and optimized MRM, DP, and CE parameters.

Data processing was performed using Analyst 1.6.3 software. A custom-built metabolite database facilitated qualitative and quantitative analyses through precise mass spectrometry. To ensure comparative accuracy between samples, systematic corrections were applied to chromatographic peaks based on retention time and peak shape characteristics, thereby enhancing the scientific validity and reliability of both qualitative and quantitative (semi-quantitative) outcomes. Quality assurance was implemented through the preparation of quality control (QC) samples—composite mixtures of equal aliquots from all sample extracts—to assess analytical reproducibility. In accordance with standardized protocols, QC samples were analyzed at regular intervals (every 10 samples) to monitor the stability and reproducibility of the analytical system continuously.

Differential metabolites were characterized using two criteria: an absolute log_2_ fold change (|log_2_(FC)| > 1) between comparative groups and variable importance in projection (VIP) values exceeding 1. Metabolite pathway enrichment analysis was performed using PlantCyc(PMN17_December2025) as the reference pathway database in Pathway Tools version 29.5 (Karp et al., 2021). Following the identification of differentially synthesized metabolites in *A. ilicifolius* from medium-salinity (M) and low-salinity (L) environments, a correlation analysis was conducted to examine the relationship between habitat salt concentration and metabolite synthesis, employing Spearman’s rank correlation coefficients. Metabolites demonstrating correlation coefficients with absolute values ≥ 0.95 (rho ≥ 0.95 or rho ≤ -0.95) were classified as key metabolites. An analogous methodological approach was employed to identify key metabolites in the high-salinity (H) and low-salinity (L) groups.

### 2.5 Identification and characterization of transcription factors associated with flavonoid pathways

First, a correlation analysis was conducted between metabolites and salt concentration in the samples using Spearman correlation analysis, with selection criteria of a correlation coefficient (rho) greater than 0.95 and a significance level of *p* < 0.05 for secondary metabolite accumulation and salt concentration. Second, using the same method, differentially expressed genes highly correlated with the salt concentration of the samples were screened. Subsequently, genes related to secondary metabolism were identified through KEGG pathway annotation, and as the accumulation of secondary metabolites progressed, Pearson correlation analysis was performed. Genes with correlation coefficients greater than 0.8 were designated as metabolite accumulation-related genes. Network diagrams were constructed using the R package ggraph (v2.2.2). Then, the same correlation analysis method was employed to select genes highly correlated with accumulated flavonoids as metabolic genes involved in flavonoid synthesis. From the differentially expressed genes, transcription factors co-expressed with these genes were screened. Correlation thresholds greater than 0.95 were employed to construct a potential transcription factor-gene pathway network diagram. Cytoscape software (v3.9.1) was used to perform correlation analysis of the regulatory relationships between DEGs and key transcription factors. The KEGG database (https://www.kegg.jp/) is utilized in this study to systematically analyze the gene information of the obtained network nodes. Based on KEGG Mapper analysis results, metabolic pathways with higher gene enrichment counts were selected for visualization in a bar chart. Meanwhile, the research focus is placed on exploring the functional mechanisms of flavonoid biosynthesis-related pathways. Networks related to flavonoid pathways were isolated from the transcription factor-gene network. To investigate the potential regulatory mechanisms underlying flavonoid biosynthesis, a flavonoid biosynthesis-related subnetwork was extracted from the transcription factor–gene correlation network. Transcription factors in this subnetwork were ranked by their RNA-seq expression levels, and the top 10% most highly expressed transcription factors were retained to construct the core regulatory network associated with flavonoid biosynthesis.

### 2.6 Verification of the relationship between transcription factors, genes, and downstream metabolites

*A. ilicifolius* plants were separated with primary roots kept intact at 30 cm length, rhizome portions preserved at approximately 30 cm, and leaves maintained in their original condition. The prepared plants were then cultivated in buckets in their native habitat. The experiment used three BEC gradient values (0.20, 0.40, and 0.60 S/m [Siemens per meter]) with a treatment duration of 5 days. After treatment, RNA was extracted using the previously described isolation method, and gene expression was verified through quantitative PCR (qPCR). Cluster-75620.679, annotated as beta-tubulin, was used as the internal reference gene for qRT-PCR normalization. Beta-tubulin has been widely used as a reference gene in plant qRT-PCR studies, including stress-related experimental conditions (Reddy et al., 2018; Li et al., 2019). The metabolite content detection followed the same process as described above.

### 2.7 Statistical analyses

All statistical analyses were conducted using R software. For O_2_^•−^ − concentration, SOD, CAT, GR, and MDA, normality and homogeneity of variance were evaluated prior to statistical testing. Data that satisfied both assumptions were analyzed using one-way ANOVA followed by Tukey’s multiple-comparison test. For data that did not meet these assumptions, the Kruskal-Wallis test was performed followed by Dunn’s multiple-comparison test with Benjamini-Hochberg adjustment. A two-sided *p* value of less than 0.05 was considered statistically significant. The criteria for identifying differentially expressed genes (DEGs) were set as follows: a significance level of *p* < 0.05 after correction by the Benjamini-Hochberg method, and a fold change in expression level of |log_2_(FC)| > 1. The screening criteria for differentially expressed metabolites (DEMs) were a significance level of *p* < 0.05 and a variable importance in projection (VIP) value greater than 1.

## 3 Results

### 3.1 Soil salinity

**Figure 1:**
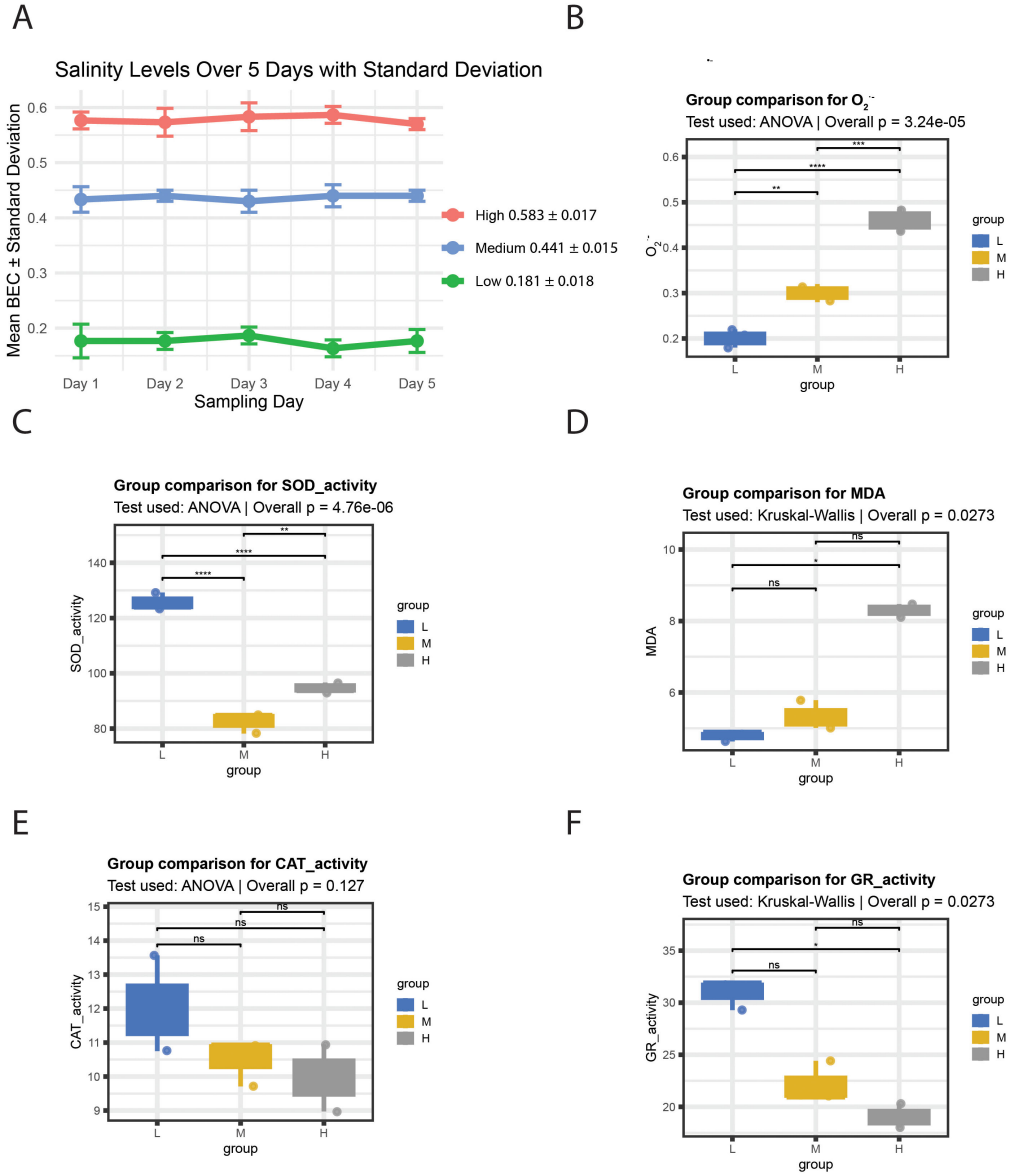
Physiological responses of *A. ilicifolius* under different salinity conditions. **(A)** Measured soil BEC across environmental salinity levels (low salt L, medium salt M, high salt H), with error bars indicating standard deviation; **(B)** Comparison of superoxide anion ( $O_{2}^{\cdot -}$) content (*μ*mol·g^−1^)showing significant increase with salinity (ANOVA, *p* = 3.2e-05); **(C)** Comparison of SOD activity (*U·*g^−1^), significantly decreased with increasing salinity (ANOVA, *p* = 4.8e-06); **(D)** Comparison of MDA content (nmol·g^−1^), significantly increased under high-salinity conditions (Kruskal-Wallis, *p* = 0.0273); **(E)** CAT activity (nmol·min^-1^·g^-1^) showed a decreasing trend with increasing salinity, though no significant difference was observed (ANOVA, *p* = 0.13); **(F)** GR activity (nmol·min^-1^·g^-1^) significantly decreased with increasing salinity (Kruskal-Wallis, *p* = 0.027). Statistical analyses were performed using one-way ANOVA, followed by Tukey’s *post hoc* test when data met the assumptions of normality and homogeneity of variance; otherwise, the Kruskal-Wallis test, followed by Dunn’s *post hoc* test with Benjamini-Hochberg correction, was used. ns, not significant; *p < 0.05; **p < 0.01; ***p < 0.001; ****p < 0.0001.

Because salinity levels significantly affect the secondary metabolites of *A. ilicifolius*, this study selected three salinity gradient sampling sites with stable salinity levels over five days. This strategic selection was implemented to control for the confounding variable of transient salinity fluctuations on the secondary metabolic processes of *A. ilicifolius*. BEC at the three selected sites was 0.18, 0.44, and 0.58 S/m. BEC showed a strong positive relationship with salinity (**Supplementary Table 1**), and the linear regression model explained 93.2% of the variance in salinity (*R*^2^ = 0.9322). The fitted equation was salinity = −0.3306 + 5.8909 × BEC, and the slope was highly significant (*p* < 2 × 10^−16^). Accordingly, the estimated salinity values at BEC levels of 0.18, 0.44, and 0.58 S/m were 0.73, 2.26, and 3.09 g/L, respectively, which categorically represent low, medium, and high salinity conditions (**Figure 1A**). These three concentration gradients solely reflect the results of our sampling within the mangrove forest. Additionally, no significant changes were observed in the phenotype of the larger plants (**Supplementary Figure 1**).

### 3.2 Analysis of antioxidant enzyme activities and markers of oxidative stress

Salt stress exposure induces excessive superoxide anion production in *A. ilicifolius* cells, as measured in *μ*mol·g^−1^ (**Figure 1B**), thereby triggering oxidative stress responses. To counteract ROS accumulation and mitigate oxidative damage to cellular membranes and organelles, plants typically activate antioxidant defense mechanisms comprising several key enzymes, including SOD, CAT, and GR. Our investigations reveal that *A. ilicifolius* exhibits atypical SOD activity (*U·*g^−1^) regulation under salt stress conditions, with a significant downregulation observed particularly in high-salinity environments (**Figure 1C**). Quantitative analyses show MDA concentrations (nmol·g^−1^), a reliable biomarker of membrane lipid degradation from lipid peroxidation, significantly increase in *A. ilicifolius* after exposure to elevated salinity beyond five days (**Figure 1D**). Furthermore, both CAT and GR enzymatic activities, expressed as nmol·min^-1^·g^-1^, exhibit pronounced inhibition under sustained high-salinity conditions (**Figures 1E, F**). CAT activity showed a decreasing trend under high salinity, although the difference was not statistically significant. The mean values and standard deviations for various salt stress treatments are presented in **Supplementary Table 3**.

### 3.3 Metabolomic responses to differential salt stress

**Figure 2:**
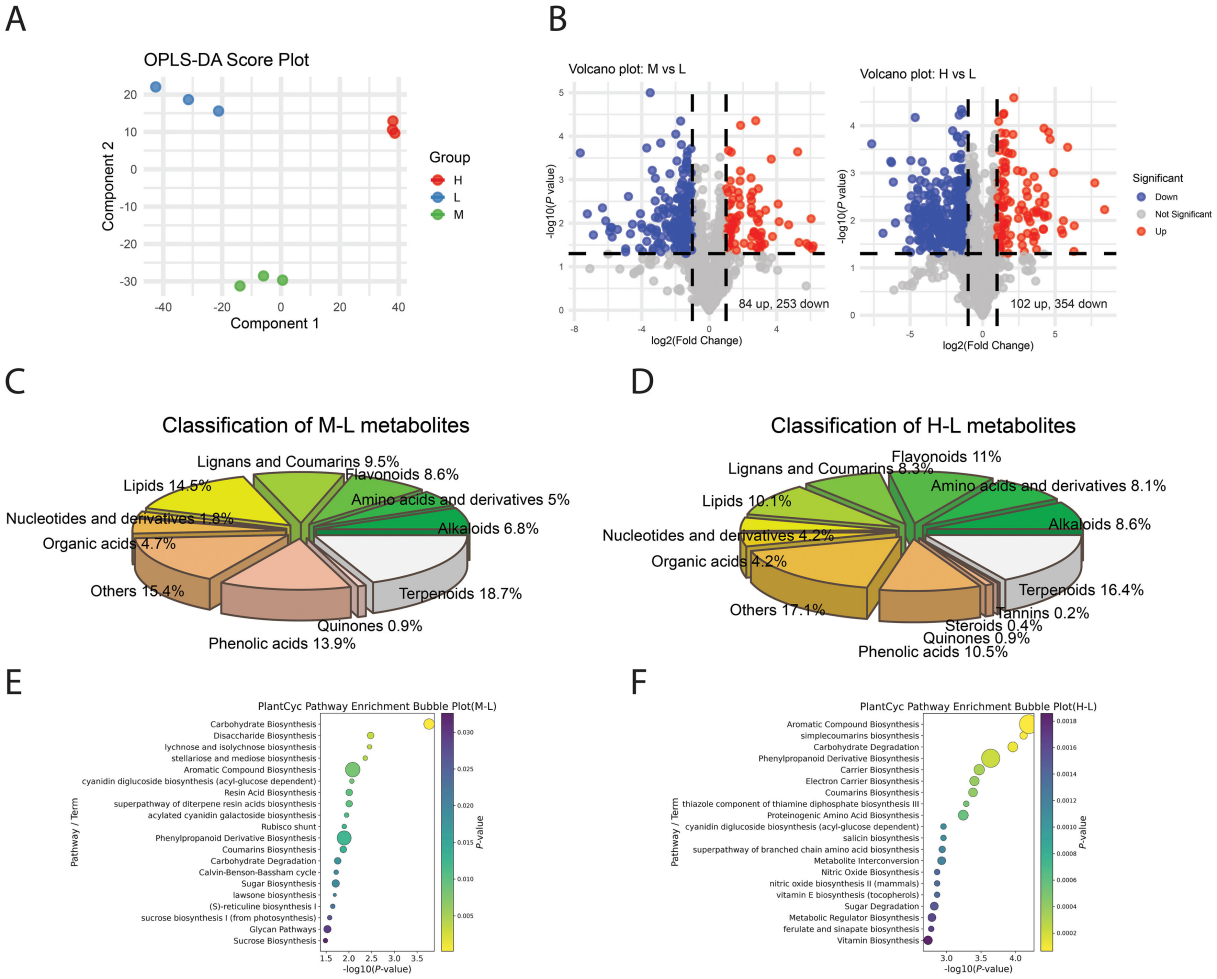
Metabolomic analysis results of *A. ilicifolius* under different salinity conditions. **(A)** OPLS-DA score plot showing the separation of metabolite profiles among high salinity (H), low salinity (L), and medium salinity (M) groups. Each point represents a sample and is colored by group; **(B)** Volcano plot comparing differentially expressed metabolites between M vs L (left) and H vs L (right) groups. Red dots indicate significantly up-regulated metabolites, blue dots indicate significantly down-regulated metabolites, and gray dots indicate metabolites with no significant difference, with 337 differential metabolites identified in M–L (84 up, 253 down) and 456 in H–L (102 up, 354 down) **(C)** Pie chart displaying the classification and relative abundance of differentially accumulated metabolites between M and L groups, highlighting major categories such as terpenoids (18.7%), lipids (14.5%), and phenolic acids (13.9%); **(D)** Pie chart displaying the classification and relative abundance of differentially accumulated metabolites between H and L groups, with major categories including terpenoids (16.4%), flavonoids (11%), and lipids (10.1%); **(E)** PlantCyc pathway enrichment analysis of the M-L comparison group. The x-axis denotes –log_10_ (*P*-value), the y-axis lists the significantly enriched pathways, bubble size reflects the number of mapped metabolites, and bubble color indicates the enrichment significance level. **(F)** PlantCyc pathway enrichment analysis of the H-L comparison group. The x-axis denotes –log_10_ (*P*-value), the y-axis lists the significantly enriched pathways, bubble size reflects the number of mapped metabolites, and bubble color indicates the enrichment significance level.

This study used broad-target metabolomics analysis, revealing notable adaptive differences in *A. ilicifolius* under three different environmental conditions (**Figure 2A**). A total of 2,151 metabolites were detected and classified into 53 metabolite categories, including glycerol esters, plumeranes, chromones, and flavonols (**Supplementary Table 4**). In the M-L comparison, 337 differential metabolites were identified, including 84 upregulated and 253 downregulated metabolites. In the H-L comparison, 456 differential metabolites were identified, including 102 upregulated and 354 downregulated metabolites. Under moderate and high salinity stress conditions, *A. ilicifolius* exhibited markedly distinct metabolite accumulation patterns (**Figure 2B**). We screened metabolites with VIP values greater than 1 and fold changes exceeding 1 for metabolite classification and pathway analysis. **Figures 2C, D** indicate that under moderate salt ion concentration stress conditions, the main metabolite categories that change in *A. ilicifolius* are terpenoids and lipids; under high salt ion concentration stress conditions, the main metabolite categories that change are flavonoids and terpenoids. The PlantCyc pathway enrichment analysis identified multiple significantly enriched pathways in both comparison groups. In the M-L group (**Figure 2E**), the top enriched pathways included carbohydrate biosynthesis, disaccharide biosynthesis, aromatic compound biosynthesis, phenylpropanoid derivative biosynthesis, rubisco shunt, carbohydrate degradation, sucrose biosynthesis, and glycan pathways. In the H-L group (**Figure 2F**), the enriched pathways were mainly aromatic compound biosynthesis, simple coumarins biosynthesis, carbohydrate degradation, phenylpropanoid derivative biosynthesis, electron carrier biosynthesis, coumarins biosynthesis, proteinogenic amino acid biosynthesis, salicin biosynthesis, nitric oxide biosynthesis, vitamin E biosynthesis, sugar degradation, and vitamin biosynthesis.

### 3.4 Transcriptomic responses to differential salt stress

After quality filtering, sequencing error-rate assessment, and GC-content distribution examination, high-quality clean reads were obtained from all nine samples for subsequent transcriptomic analysis. The number of clean reads ranged from 43,695,662 to 72,190,476 among the samples, indicating that the sequencing depth was sufficient for downstream analysis. *De novo* assembly using Trinity generated 326,806 transcripts and 197,038 unigenes. Principal component analysis (PCA) was further performed to evaluate the overall variation among samples (**Supplementary Figure S2**). The PCA results showed clear separation among the low-, medium-, and high-salinity groups, while biological replicates within each treatment clustered relatively closely, suggesting good reproducibility among replicates and distinct transcriptomic responses to different salinity conditions. These results support the reliability of the transcriptomic dataset for subsequent differential expression and functional enrichment analyses.

**Figure 3:**
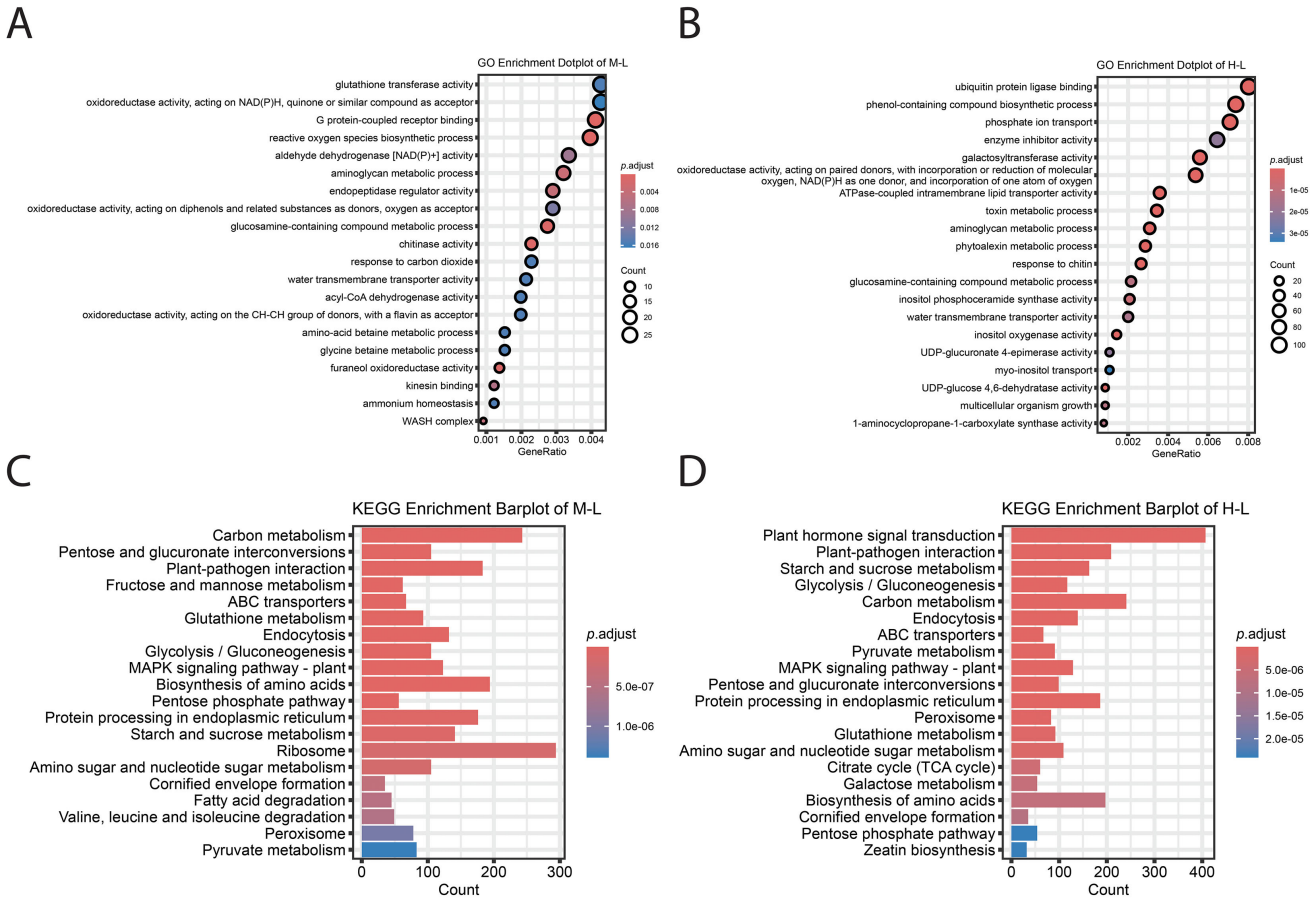
GO enrichment analysis and KEGG pathway enrichment analysis of differentially expressed genes. **(A)** GO enrichment scatter plot of genes differentially expressed between medium-salt (M) and low-salt (L) treatments. The x-axis represents gene proportion, dot size reflects gene quantity, and color indicates significance level of corrected *p*-values. Major enrichments occurred in functional categories such as oxidoreductase activity, metabolic processes, and transporter activity. **(B)** GO enrichment scatter plot for differentially expressed genes between high-salt (H) and low-salt (L) treatments. This plot highlights key biological process and molecular function enrichments, including phenolic compound biosynthesis, transporter activity, and metabolic processes related to inositol and glucosamine. **(C)** KEGG pathway enrichment bar chart for the M-L group, displaying gene counts associated with significantly enriched pathways. Major pathways include carbon metabolism, plant-pathogen interactions, glutathione metabolism, glycolysis/gluconeogenesis, and peroxisome function. **(D)** KEGG pathway enrichment bar chart for the H-L group, displaying major enriched pathways such as plant hormone signaling, starch and sucrose metabolism, glycolysis/gluconeogenesis, MAPK signaling, peroxisomes, and auxin biosynthesis. Bar colors represent corrected *p*-values, indicating the significance levels of pathway enrichment.

The gene expression patterns of *A. ilicifolius* exhibited significant variations under different salt stress conditions. In the medium salt concentration stress group, transcriptome analysis identified 6,729 upregulated genes and 3,603 downregulated genes. Conversely, the high salt concentration stress group demonstrated 5,047 upregulated genes, while the number of downregulated genes increased substantially to 16,636. Gene Ontology (GO) enrichment analysis revealed three significantly enriched GO annotation entries shared between the M group and H group in response to salt stress: glucosamine-containing compound metabolic process, aminoglycan metabolic process, and water transmembrane transporter activity. Notably, oxidoreductase activity-related genes in the M group primarily involved NAD(P)H-related metabolic processes, flavin compounds, and furaneol, whereas those in the H group were predominantly associated with phenol and inositol. KEGG pathway analysis demonstrated considerable consistency in enriched pathways. The metabolic pathways affected by RNA gene expression alterations in the transcriptome showed high consistency across different salt concentration stress levels, particularly among the top 20 statistically significantly enriched pathways (**Figures 3C, D**). These pathways include Glutathione metabolism and Peroxisome pathways, which are directly implicated in antioxidant activity.

### 3.5 Key metabolic products and gene expression analysis

**Figure 4:**
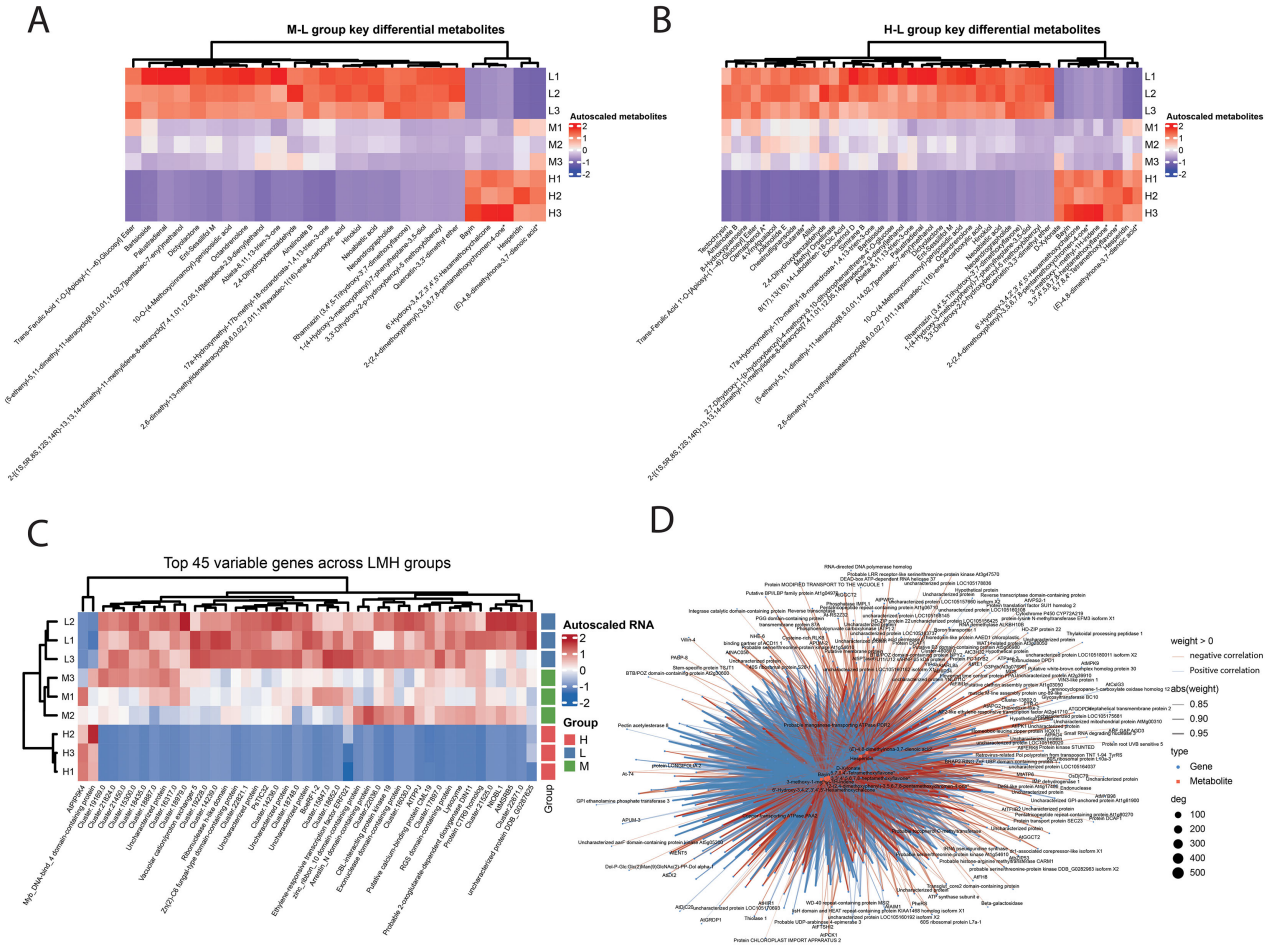
Transcriptional factor expression analysis and metabolic association network. **(A, B)** Heatmaps showing changes in metabolites across different salinity treatments (low salt L, medium salt M, and high salt H). Red indicates upregulation, blue indicates downregulation. **(C)** Heatmap showing gene changes across different salinity treatments, with only 25 randomly selected genes depicted. **(D)** Correlation network between differentially expressed genes and differentially accumulated secondary metabolites. Blue circles denote genes and orange squares denote differential metabolites; edge color indicates correlation direction (blue = positive, red = negative) and edge thickness is scaled by absolute correlation magnitude.

In the M-L group, Bayin, Hesperidin, 6’-Hydroxy-3,4,2’,3’,4’,5’-Hexamethoxychalcone, and (*E*)-4,8-dimethylnona-3,7-dienoic acid were increased (**Figure 4A**). The synthesis of these four substances showed a correlation higher than 0.95 with the salinity level at which the plants were located. Interestingly, in addition to the 4 substances mentioned above, the H-L group also accumulated more D-Xylonate, 3,3’,4’,5,6,7,8-heptamethoxyflavone, and 5,7,8,4’-Tetramethoxyflavone et al. (**Figure 4B**). In habitats with different salt concentrations, 10416 genes with R values greater than 0.8 for salt were screened. Among them, 8 genes are annotated as SOD or CAT (**Supplementary Table 5**). The top 45 genes with the greatest expression variability across the low-, medium-, and high-salinity groups were selected to generate the heatmap (**Figure 4C**). Additionally, 702 genes are associated with the synthesis of secondary metabolites (**Supplementary Table 6**). For example, Cluster-18867.0 is linked to flavonoid biosynthesis, while Cluster-15415.0 is part of the peroxisome pathway. To determine which accumulated secondary metabolic components correlate with gene expression under increasing salt concentrations, we identified 650 key genes with R values greater than 0.8 for accumulated secondary metabolites (**Figure 4D**; **Supplementary Table 7**).

**Figure 5:**
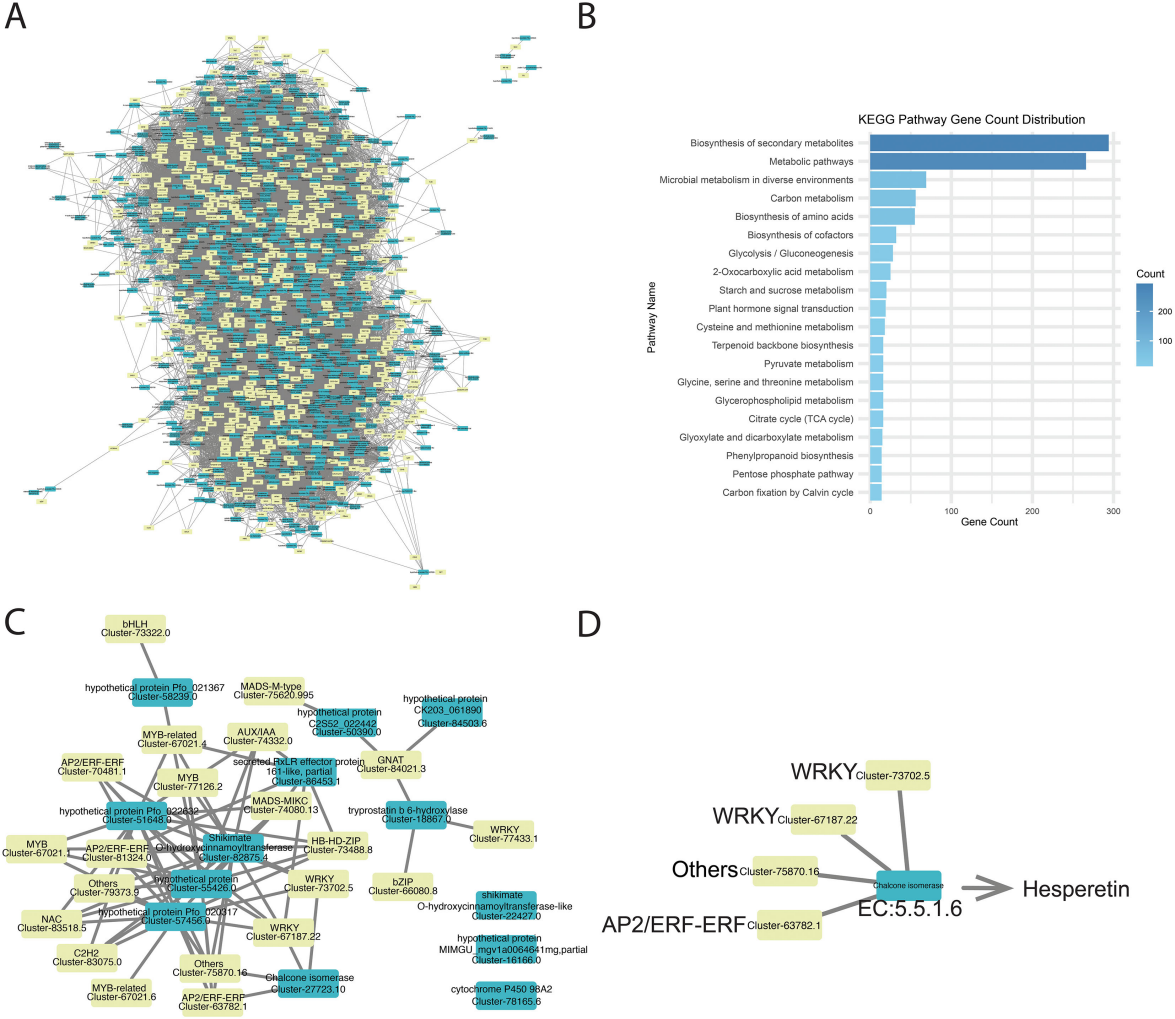
Identification of candidate transcription factors associated with flavonoid accumulation. **(A)** Overview of the correlation network between candidate transcription factors and flavonoid-associated DEGs linked to the accumulation of six differential flavonoids. Yellow nodes represent candidate transcription factors, whereas blue nodes represent flavonoid-associated DEGs whose expression levels were highly correlated with the six flavonoids. The flavonoid-associated DEG set was not restricted to flavonoid biosynthetic structural genes and may also include transcription factors. **(B)** Top 20 enriched pathways after mapping all network genes to the KEGG map. **(C)** Network of differentially expressed genes in the flavonoid metabolic pathway and their associated transcription factors. Yellow indicates potential transcription factor types; blue indicates genes mappable to the flavonoid metabolic pathway. **(D)** Transcriptional factors and metabolites highly correlated with Cluster-27723.10.

**Figure 6:**
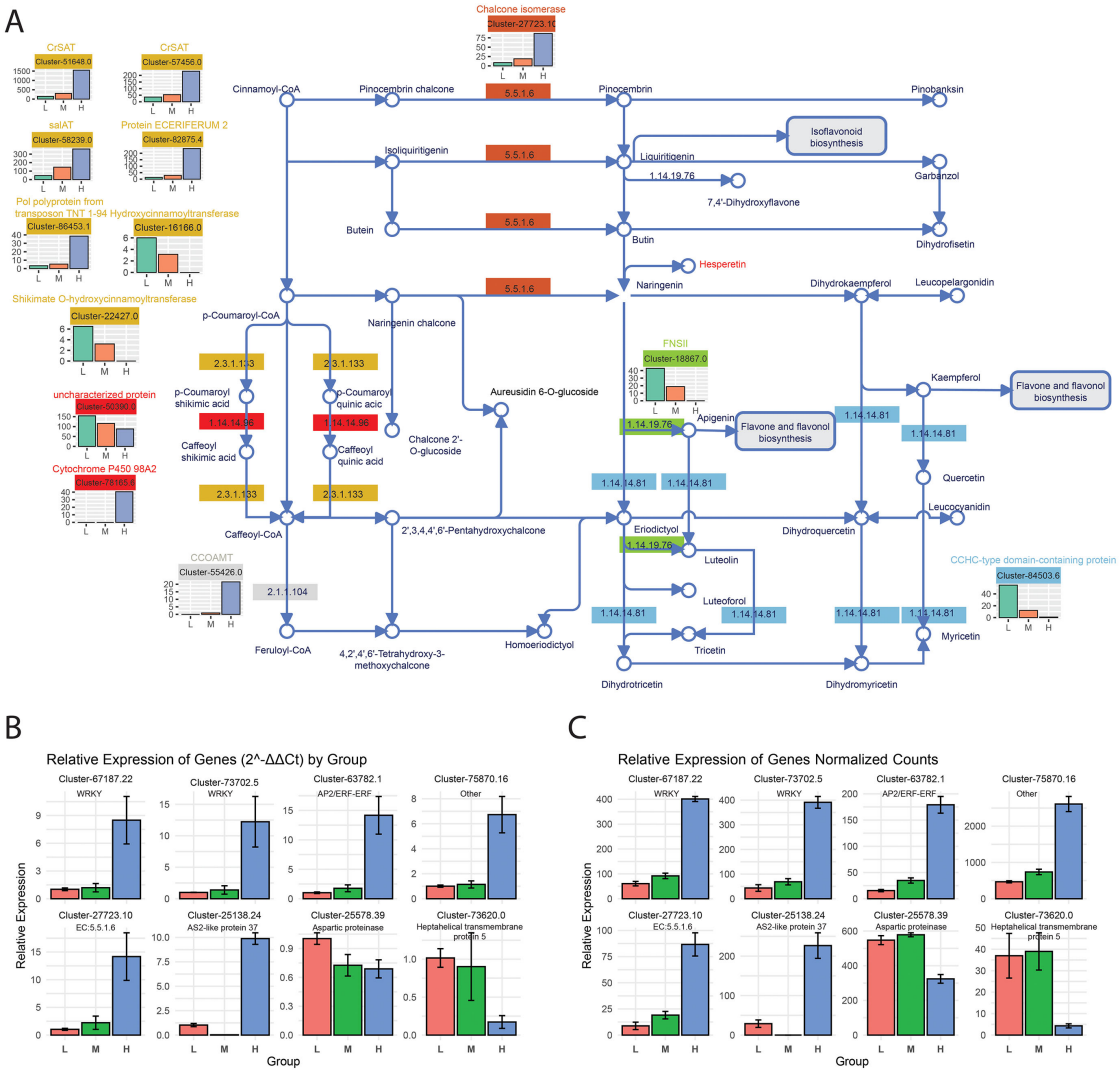
Hesperetin synthesis pathway analysis. **(A)** Expression levels of various gene types in the flavonoid biosynthesis pathway. Numbers within the pathway represent enzyme EC numbers; genes with matching colors are annotated as encoding that enzyme. **(B)** qPCR validation of selected candidate genes in *A. ilicifolius* under different BEC gradients. Cluster-75620.679 (annotated as Tubulin beta-2 chain) was used as the internal reference gene and was set to 1 for normalization across all groups. Four annotated genes were selected from the candidate regulatory network shown in **Figure 5D**, associated with EC 5.5.1.6-related processes and hesperetin biosynthesis. Gene IDs with corresponding annotations or gene-family information are shown in the figure. Additionally, three genes (Cluster-25138.24, Cluster-25578.39, and Cluster-73620.0) were amplified from the same RNA-seq sample. **(C)** Bar chart showing sequencing counts for *A. ilicifolius* samples collected at different salt concentrations.

A subnetwork of flavonoids was extracted in **Figure 4D**. These flavonoids include Hesperidin, Bayin, 2-(2,4-dimethoxyphenyl)-3,5,6,7,8-pentamethoxychromen-4-one, 3,3’,4’,5,6,7,8-heptamethoxyflavone, 6’-Hydroxy-3,4,2’,3’,4’,5’-Hexamethoxychalcone, and 5,7,8,4’-Tetramethoxyflavone. After identifying 616 flavonoid-associated DEGs that were positively or negatively correlated with differential flavonoid metabolites, we further assessed their associations with 564 transcription factors (TFs) among the 2,042 TF-annotated genes. TF–DEG pairs with an absolute correlation coefficient of *r* ≥ 0.95 were retained to identify candidate TFs potentially associated with flavonoid accumulation (**Figure 5A**). The NR and TF-Family annotation results for all genes are presented in **Supplementary Table 8**. These genes and their associated transcription factors are involved in fundamental metabolic pathways, including glycolysis/gluconeogenesis, the TCA cycle, lipid metabolism, and amino acid metabolism (**Figure 5B**). It was found that these differentially expressed genes could be annotated to pathways in flavonoid metabolism, so their gene-TF network was isolated from the leading network (**Figure 5C**). For subsequent validation, only the top 10% of highly expressed transcription factors were kept. The positions of differentially expressed genes within flavonoid biosynthesis are shown in **Figure 6A**. The figure reveals that hesperidin accumulation in flavonoid biosynthesis is correlated with EC 5.5.1.6. While this enzyme is classified as a hypothetical protein in NR data, it is identified as a chalcone-flavonone isomerase-like isoform X1 in KEGG data. Four transcription factors associated with EC:5.5.1.6 were therefore isolated from the gene-transcription factor network (**Figure 5D**). Two belong to the WRKY family. In contrast, the other two are classified as AP2/ERF-ERF and Others, respectively.

In the validation experiment, the relation between AP2/ERF-ERF, WRKY transcription factors, and their target genes was evaluated across a gradient of BEC values (0.20, 0.40, 0.60), with the results being presented in **Figures 6B, C**. Elevated expression levels under high-salt conditions were demonstrated by all three transcription factors in the validation experiments, which corroborates our sequencing data. The EC 5.5.1.6 enzyme gene also exhibited similar expression patterns. Importantly, hesperidin was confirmed to be significantly enriched at all tested BEC gradient values (0.40 and 0.60) by our metabolite analysis, as illustrated in **Supplementary Figure 3**. However, the qPCR validation results showed that, among the nine randomly selected genes, most exhibited expression trends consistent with the transcriptomic data, whereas Cluster-25578.39 displayed an opposite expression pattern. Overall, the qPCR validation was broadly consistent with the transcriptomic data, except for Cluster-25578.39, which showed a divergent expression pattern (**Figure 6**; **Supplementary Figure S4**).

## 4 Discussion

### 4.1 Salinity-induced oxidative stress and the atypical decline in SOD activity

The use of three field sites with relatively stable salinity levels minimized the influence of transient salinity fluctuations and enabled a clearer evaluation of the physiological response of *A. ilicifolius* to different salt environments. The physiological data showed that increasing soil salinity, reflected by BEC values, progressively elevated intracellular ROS levels and imposed substantial oxidative stress on plant tissues. This pattern is consistent with the general view that salinity disrupts redox homeostasis and challenges antioxidant capacity in plants (Akhtar et al., 2026).

Under salt stress, antioxidant enzymes typically provide the first line of defense. SOD is a key metalloenzyme responsible for the dismutation of superoxide radicals and is often induced under saline conditions in both mangrove and non-mangrove species. For example, increased SOD activity has been reported in *Sonneratia alba* and *Bruguiera gymnorrhiza* under high salinity (Takemura et al., 2002; Yang et al., 2016). By contrast, *A. ilicifolius* showed a significant decline in SOD activity under severe salinity, indicating that its antioxidant response differs from the more typical halophytic pattern.

One plausible explanation is that excessive ROS accumulation under high salinity exceeded the buffering capacity of the enzymatic antioxidant system, leading not only to cellular oxidative damage but also to direct impairment of antioxidant enzymes. The concomitant increase in MDA and the decline in CAT and GR activities support this interpretation, suggesting that oxidative stress progressed to membrane lipid peroxidation and broader enzymatic dysfunction. In addition, although intracellular ion concentrations were not measured, salinity-induced ionic imbalance may have affected the availability or utilization of essential SOD cofactors such as Cu, Zn, and Mn.

More importantly, the decline in SOD activity may reflect a shift in defense strategy rather than a simple collapse of protection. Under extreme salinity, maintaining energetically costly enzyme-based detoxification may become less efficient, favoring the accumulation of low-molecular-weight non-enzymatic antioxidants. In this context, the parallel increase in flavonoid-related metabolites suggests that *A. ilicifolius* partially compensates for weakened enzymatic defenses by reinforcing chemical ROS scavenging. This interpretation is supported by the transcriptomic data: CAT activity closely tracked the down-regulation of CAT-related genes, whereas changes in total SOD activity were only partly reflected by the expression patterns of individual SOD isoforms (**Figure 1**; **Supplementary Table S5**). Together, these observations suggest that high salinity disrupts ROS homeostasis in *A. ilicifolius* through both transcriptional suppression and functional impairment of antioxidant enzymes, thereby necessitating alternative antioxidant strategies.

### 4.2 Systematic comparison of terpenoid and secondary metabolite responses in mangroves

Broad-spectrum metabolomics revealed a clear salinity-dependent shift in secondary metabolism in *A. ilicifolius*. Under moderate salinity, the major metabolic changes involved terpenoids, lipids, phenolic acids, and lignans/coumarins, suggesting that the early response primarily involved membrane remodeling and adjustment of basal protective metabolism. Under high salinity, however, the relative contribution of flavonoids increased from 8.6% to 11.0%, whereas lipids declined and terpenoid dominance weakened (**Figures 2C, D**). This shift suggests that increasing salinity progressively redirected metabolism from structural adjustment toward chemically active antioxidant defenses.

The PlantCyc enrichment results further support this interpretation. Under high salinity, the enrichment of aromatic compound biosynthesis and phenylpropanoid derivative biosynthesis indicates enhanced flux into pathways supplying precursors for flavonoids, coumarins, and other phenolic defense compounds (**Figures 2E, F**). The concurrent enrichment of carbohydrate degradation suggests active carbon mobilization to sustain this reprogramming. Taken together, these data indicate that high salinity promoted not merely metabolic disturbance, but a directed reallocation of carbon and precursor supply toward phenylpropanoid-derived antioxidant metabolism.

Comparison with other mangrove species highlights the distinctiveness of this response. In non-salt-secreting mangroves such as *Kandelia candel* and *Bruguiera gymnorrhiza*, salt stress has been associated with increased triterpenoid accumulation, particularly in roots, together with upregulation of key triterpenoid biosynthetic genes such as triterpene synthase, lupeol synthase, and *β*-amyrin synthase (Takemura et al., 2002; Tada and Kashimura, 2009; Nizam et al., 2024). In these species, terpenoid accumulation likely contributes to membrane stabilization and reduced ion permeability. By contrast, although *A. ilicifolius* also altered terpenoid metabolism, its dominant response under severe salinity appeared to shift toward flavonoid accumulation.

This distinction may reflect differences in adaptive strategy among mangroves. As a salt-secreting species, *A. ilicifolius* can regulate ion balance through salt glands. However, once external salinity exceeds the buffering capacity of this anatomical system, biochemical ROS detoxification may become more important than further strengthening membrane barriers. In this context, the increased reliance on flavonoids is biologically plausible, because flavonoids are recognized as major non-enzymatic antioxidants in plant salt stress responses. Previous work in *Acanthus* species has likewise suggested that flavonoids contribute to stress resistance under saline conditions (Parida et al., 2004). Thus, the contrast between a flavonoid-centered response in *A. ilicifolius* and a more triterpenoid-oriented response in other mangroves suggests divergent strategies of salt adaptation, with some species emphasizing structural ion exclusion and others relying more strongly on biochemical antioxidant compensation.

### 4.3 Transcriptional coordination of the salt stress response

The GO and KEGG analyses suggest that the metabolic shift toward flavonoid accumulation was supported by coordinated transcriptional regulation. Under moderate salinity, enriched GO terms were mainly associated with glutathione transferase activity, oxidoreductase activity, reactive oxygen species biosynthetic process, and aldehyde dehydrogenase activity (**Figure 3A**), indicating that the early response of *A. ilicifolius* centered on ROS regulation, detoxification, and redox homeostasis. Under high salinity, the enrichment pattern extended beyond general stress-response functions to include phenol-containing compound biosynthetic process and phytoalexin metabolic process (**Figure 3B**). Because flavonoids are major phenolic secondary metabolites, and phytoalexin-related metabolism is typically associated with defense activation, this transition suggests that the response to severe salinity shifted from predominantly enzymatic redox adjustment toward stronger induction of phenolic secondary metabolism.

KEGG enrichment supports the same interpretation from a pathway perspective. Under moderate salinity, enrichment of carbon metabolism, pentose and glucuronate interconversions, starch and sucrose metabolism, glycolysis/gluconeogenesis, the pentose phosphate pathway, and glutathione metabolism indicates substantial reprogramming of carbon allocation and redox buffering (**Figure 3C**). These pathways can provide carbon skeletons, ATP, and reducing power for phenylpropanoid and flavonoid biosynthesis. Under high salinity, the additional enrichment of plant hormone signal transduction, plant-pathogen interaction, and the MAPK signaling pathway, together with persistent enrichment of carbon metabolism and glutathione-related pathways, points to an integrated response involving stress signaling, metabolic redistribution, and antioxidant regulation (**Figure 3D**). This pattern is consistent with current models in which salt-induced flavonoid accumulation is embedded in broader networks linking redox homeostasis, signaling, and secondary metabolism (Akhtar et al., 2026).

Although flavonoid biosynthesis did not emerge as one of the most prominent GO or KEGG categories, this does not diminish its biological relevance. When interpreted together with the metabolomic data, the increase in flavonoid abundance from 8.6% under moderate salinity to 11.0% under high salinity, together with the enrichment of phenylpropanoid-related precursor pathways, strongly suggests that flavonoid accumulation was a meaningful component of the high-salinity response rather than a passive by-product. Thus, the transcriptomic data support a model in which oxidative stress, glutathione-associated redox buffering, carbon reallocation, and stress signaling collectively create a regulatory environment favorable for flavonoid biosynthesis in *A. ilicifolius*.

### 4.4 Integration of transcriptomic and metabolomic reprogramming strategies

Integrated analysis of the metabolomic and transcriptomic datasets further clarified how flavonoid accumulation was linked to salinity-dependent gene regulation. In the M-L comparison, Bayin, Hesperidin, 6’-Hydroxy-3,4,2’,3’,4’,5’-hexamethoxychalcone, and (*E*)-4,8-Dimethylnon-3,7-diene acid were positively associated with salinity (**Figure 4A**), whereas under high salinity, additional metabolites such as D-xylonate, 3,3’,4’,5,6,7,8-heptamethoxyflavone, and 5,7,8,4’-tetramethoxyflavone accumulated further (**Figure 4B**). The predominance of flavonoid-related metabolites in this set supports the view that flavonoid accumulation scaled with salt intensity and therefore likely contributed to adaptation rather than representing a coincidental metabolic shift.

At the transcriptional level, salinity-responsive genes included components associated with antioxidant defense as well as secondary metabolism (**Figure 4C**, **Supplementary Tables 5**, **6**), indicating that oxidative stress adaptation and metabolite reprogramming were tightly coupled. Correlation-based network analysis further identified a flavonoid-associated subnetwork containing hesperidin, Bayin, methoxylated flavones, and chalcone-related compounds, together with 616 positively or negatively associated genes (**Figure 4D**). The associated transcription factors and candidate genes were connected not only to secondary metabolism but also to glycolysis/gluconeogenesis, the TCA cycle, lipid metabolism, and amino acid metabolism (**Figures 5A, B**, **Supplementary Table 8**), indicating that flavonoid accumulation under high salinity was supported by system-wide metabolic reorganization.

A particularly important finding was the identification of a putative regulatory node linking hesperidin accumulation to EC 5.5.1.6, which was annotated in KEGG as a chalcone-flavanone isomerase-like isoform X1 (**Figure 6A**). Although the NR annotation classified this gene as a hypothetical protein, its KEGG identity is mechanistically plausible because chalcone isomerase-related reactions occupy a central position in the early flavonoid pathway and influence the formation of downstream flavanones and their derivatives. This node was further connected to four candidate transcription factors, including two WRKY members, one AP2/ERF-ERF factor, and one factor annotated as Others (**Figure 5D**). Given the established roles of WRKY and AP2/ERF family members in stress signaling and flavonoid regulation, these candidates provide a plausible regulatory framework for salinity-induced flavonoid biosynthesis in *A. ilicifolius*.

The validation data support this framework. Across the BEC gradient, WRKY and AP2/ERF-ERF transcription factors, together with the EC 5.5.1.6-related gene, were all upregulated under high-salt conditions (**Figure 6B, C**), and hesperidin remained significantly enriched at BEC values of 0.40 and 0.60 (**Supplementary Figure S2**). Although one randomly selected gene showed an inconsistent qPCR trend, most validation results were consistent with the transcriptomic dataset (**Figure 6**; **Supplementary Figure S3**), supporting the robustness of the inferred regulatory module. Collectively, these data support a model in which increasing salinity elevates oxidative stress, activates WRKY- and AP2/ERF-associated regulation, promotes chalcone-flavanone isomerase-related activity, and ultimately drives the accumulation of hesperidin and related flavonoids.

Overall, *A. ilicifolius* appears to cope with high salinity by shifting from an increasingly compromised enzymatic antioxidant system toward a flavonoid-centered chemical defense strategy. This transition links oxidative stress perception, transcriptional control, and secondary metabolite accumulation into a coherent adaptive framework, and may also help explain the medicinal relevance of this species through the enrichment of bioactive flavonoid-related compounds.

### 4.5 Limitations and future perspectives

While this study provides valuable insights into the adaptive mechanisms of *A. ilicifolius* under salt stress, several limitations should be acknowledged.

First, regarding study design and physiological measurements, our findings are based on a one-time assessment of field-observed external salinity gradients. Furthermore, we must acknowledge a potential limitation regarding sample independence. Due to the dense natural growth habit of *A. ilicifolius*, the spatial proximity of sampled individuals may inadvertently introduce microenvironmental biases or pseudo-replication, which could potentially inflate statistical significance and limit the generalizability of the results. Future studies should incorporate strict spatial separation protocols, alongside multi-seasonal and tidal-cycle monitoring, to better understand natural salinity dynamics. To confirm the transmission of salt stress into plant tissues, these field observations must be complemented by controlled salt-stress experiments that include systematic phenotypic imaging and direct measurements of internal tissue Na^+^/K^+^ ratios and Cl^-^ contents.
Second, regarding oxidative damage and enzyme regulation, our observations of ROS accumulation and lipid peroxidation suggest membrane-level oxidative stress, but without direct microscopic observation of cellular damage or electrolyte leakage assays, these results should not be overinterpreted as irreversible cellular injury. Furthermore, the discrepancies between antioxidant gene expression and SOD/CAT activities imply complex post-transcriptional or post-translational regulatory processes. Future research requires isoform-specific enzyme assays, protein-level validation, and subcellular localization to clarify these dynamics.
Finally, regarding gene expression validation and regulatory mechanisms, the use of Cluster-75620.679 as the qRT-PCR reference was based on its beta-tubulin annotation and previous literature support, rather than on a systematic stability-ranking analysis in this study. Although this choice allowed us to proceed with expression validation, it may have introduced some uncertainty into the interpretation of the qRT-PCR results. Therefore, these results should be regarded as supportive evidence for the transcriptomic data rather than definitive validation. In addition, the inferred TF regulatory networks and the association between EC 5.5.1.6 and hesperidin biosynthesis were derived from co-expression correlations rather than direct causal evidence. Future studies will require targeted metabolomics and functional validation, including yeast one-hybrid assays, EMSA, ChIP-seq, and metabolic flux analysis, to confirm direct TF–target interactions and downstream enzymatic reactions.

## 5 Conclusion

**Figure 7:**
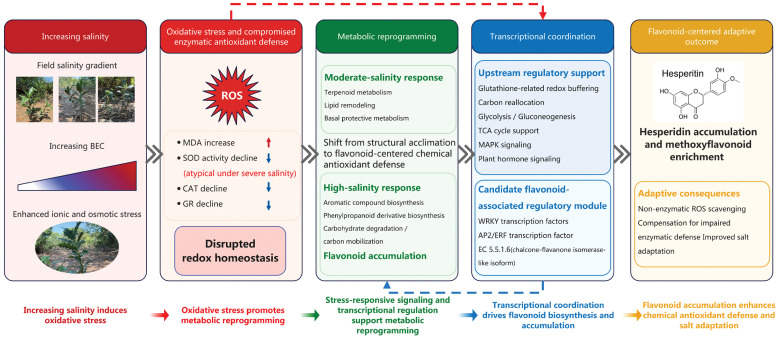
Integrative model of transcriptomic and metabolomic reprogramming in Acanthus ilicifolius under salinity stress. Increasing salinity induces oxidative stress and impairs enzymatic antioxidant defense, leading to disrupted redox homeostasis. Integrated metabolomic and transcriptomic analyses indicate a salinity-dependent shift from terpenoid/lipid-associated metabolism under moderate salinity to phenylpropanoid- and flavonoid-centered metabolism under high salinity, together with stress-responsive signaling and candidate WRKY/AP2/ERF-associated regulation. This reprogramming promotes hesperidin and methoxyflavonoid accumulation, which likely compensates for weakened enzymatic defenses through enhanced non-enzymatic ROS scavenging.

In conclusion, this study integrated transcriptomic and metabolomic analyses to elucidate the physiological and molecular responses of *A. ilicifolius* across a field-observed salinity gradient (**Figure 7**). Our findings reveal that high salinity stress compromises the traditional enzymatic antioxidant defense system, evidenced by decreased SOD, CAT, and GR activities alongside elevated ROS and lipid peroxidation. However, rather than indicating irreversible cellular injury, this points to a vulnerability that triggers a distinct adaptive strategy: a shift from enzymatic to non-enzymatic biochemical defenses. *A. ilicifolius* exhibits significant metabolic reprogramming depending on stress intensity. While moderate salinity primarily modulates lipid and terpenoid pathways, high salinity drives a substantial accumulation of flavonoids, such as hesperidin, suggesting a strategic diversion of carbon metabolism toward secondary antioxidant compounds. Furthermore, co-expression networks identified transcription factors from the WRKY and AP2/ERF families as candidate regulators potentially coordinating this flavonoid biosynthesis. Although the precise causal relationships of this metabolic rerouting require further functional validation through controlled experiments, our study provides crucial insights into the salt tolerance mechanisms of *A. ilicifolius*.

## Data Availability

The datasets presented in this study can be found in online repositories. The names of the repository/repositories and accession number(s) can be found below: https://github.com/pdpmb/Acanthus.

## Supplementary material

Supplementary Figure 1 Field environments and corresponding BEC values at different sampling sites. **(A)** Sampling site A, BEC = 0.58 S/m; **(B)** Sampling site B, BEC = 0.44 S/m; **(C)** Sampling site C, BEC = 0.18 S/m.

Supplementary Figure 2 Principal component analysis (PCA) of transcriptomic profiles from *A. ilicifolius* under low-, medium-, and high-salinity treatments. Each point represents a biological replicate. The clear separation of the three salinity groups along PC1 and PC2 reflects distinct global transcriptomic responses to increasing salinity, whereas the tight clustering of replicates within each group indicates high intra-group consistency and supports the robustness of the RNA-seq data.

Supplementary Figure 3 Bar chart of hesperidin content and its standard error (Mean ± SE) under different BEC conditions. The figure shows that Hesperidin content significantly increases as the BEC value rises from 0.20 to 0.60, with error bars representing the standard error for each group.

Supplementary Figure 4 Validation of RNA sequencing data. **(A)** qPCR results for six randomly selected genes in *A. ilicifolius*, obtained from BEC values of 0.18, 0.44, and 0.58 S/m at different sites to verify RNA sequencing accuracy. Cluster-75620.679 was used as the internal control gene. **(B)** Bar graphs displaying the normalized read counts for these genes across *A. ilicifolius* samples collected at various salt concentrations.
